# Trends in repeated pregnancy among adolescents in the Philippines from 1993 to 2013

**DOI:** 10.1186/s12978-018-0630-4

**Published:** 2018-11-06

**Authors:** Joemer C. Maravilla, Kim S. Betts, Rosa Alati

**Affiliations:** 1 School of Public Health, The University of Queensland, Herston, QLD Australia; 2 Institute for Social Science Research, The University of Queensland, Indooroopilly, QLD Australia; 3 Centre for Youth Substance Abuse Research, The University of Queensland, Herston, QLD Australia

## Abstract

**Objective:**

The extent of repeated pregnancy (RP) and repeated birth (RB) among adolescents aged 15–19 is still unknown in the Philippines despite the health and socio-economic consequences. This study aims to investigate the RP and RB prevalence trends in the Philippines from 1993 to 2013.

**Methods:**

A total of 7091 women aged 15–24 who experienced at least one pregnancy were captured in the Philippine demographic health surveys from 1993 to 2013. Annual RP and RB prevalence per age group in three and five categories were calculated and stratified by region, type of residence and wealth index. Cochran–Armitage tests and multivariate logistic regression were applied to determine trend estimates.

**Results:**

Compared to women aged 19–21 years and 22–24 years, for which decreasing patterns were found, RP ([Adjusted Odds ratio (AOR =0.96; 95%Confidence interval (CI) =0.82–1.11) and RB (AOR = 0.90; CI = 0.73–1.10) trends among 15–18 year olds showed negligible reduction over the 20 years. From a baseline prevalence of 20.39% in 1993, the prevalence of RP among adolescents had only reduced to 18.06% by 2013. Moreover, the prevalence of RB showed a negligible decline from 8.49% in 1993 to 7.80% in 2013. Although RP and RB prevalence were generally found more elevated in poorer communities, no differences in trends were noted across wealth quintiles.

**Conclusion:**

For two decades, the Philippines has shown a constant and considerably high RP prevalence. Further investigation, not only in the Philippines but also in other developing countries, is necessary to enable development of secondary prevention programs.

## Plain English summary

Despite high and stable levels of adolescent fertility in the Philippines, no specific research has been conducted to specifically measure the trend and magnitude of repeated adolescent pregnancy, which is defined as an adolescent who has had at least two pregnancies. Repeated pregnancy, therefore needs to be investigated as it reflects not only the reproductive health of adolescent mothers but also disparities in service delivery of health, education and welfare support to adolescents after their first pregnancy.

We used the Philippine Demographic and Health Surveys to sample 7091 women aged 15–24 who experienced at least one pregnancy. Annual RP and RB prevalence per age group in three and five categories were calculated and stratified by region, type of residence and wealth quintile. Trends were statistically analysed using Cochran–Armitage tests and multivariate logistic regression.

While a decline was observed in 19–21 and 22–24 year olds, we found a constant prevalence of one in every five in 15–18 years old from 1993 to 2013. This trend was evident across all regions, types of residence and socio-economic status. Our analysis also found that those from the poorest wealth quintile demonstrated a heightened risk of repeated pregnancy compared to other quintiles. The non-decreasing prevalence trend of repeated pregnancy among adolescents indicated the need for secondary prevention programs particularly for the poorest households. Epidemiological investigations are also necessary to explore the causes and impact of repeated pregnancy on maternal, child and neonatal health, not only in the Philippines, but also among other low- and middle-income countries.

## Introduction

The adolescent pregnancy epidemic in the Philippines has been acknowledged as one of the worst in the Western Pacific Region [1] with a recent prevalence of 13.6% among 15–19 year olds. The Philippines is the only country in this region with no significant decline in adolescent fertility in the past decades [2] from 56 per 1000 in 1973 to 57 per 1000 in 2013 [2, 3]. In order to address this entrenched public health issue, preventive policies and programs have been implemented [4, 5], and epidemiological studies have been developed to provide evidence of the current sexual health and behaviour of Filipino adolescents [6]. However, these measures have put little emphasis on the more serious problem of repeated adolescent pregnancies.

Repeated adolescent pregnancy, which is defined as a subsequent pregnancy among adolescents aged 10–19 years [7] is known to affect around 18% of adolescent mothers in the USA [7], Europe [8], and Australia [9]. Despite the evident chance of repeated adolescent pregnancy especially within 2 years postpartum [10], current research is unable to clearly establish its magnitude in developing countries such as the Philippines, nor how the trends have changed across time [11–13]. Although a World Health Organization (WHO) multi-country report [14] discussed the relationship between age and parity among Filipino adolescents, this study did not assess the prevalence of multi parity as its primary measure.

As a marker for adolescent reproductive health, repeated pregnancy reflects health disparities particularly among the disadvantaged adolescent population. Repeated pregnancy also indicates poor distribution and unequal access to reproductive health services [15] and inadequate service capacity of individual localities. It relates to low educational attainment, limited employment opportunities and poverty among adolescent mothers [15, 16]. It has been shown that repeated adolescent pregnancy leads to an increase in national health and welfare expenditure as a consequence of the long-term dependency of adolescents and their families on government assistance [15, 17].

An increasing trend of adolescent sexual activity [3] ongoing poor compliance with modern contraceptives [2, 18] and inadequate use of family planning services all suggest that repeated adolescent pregnancy is highly prevalent in the Philippines [12]. Analysis of existing nationally representative data can be helpful in evaluating the extent of this public health problem. In this study, we aim to determine the prevalence of repeated pregnancies and births among adolescents and young adults from a series of national surveys conducted between 1993 and 2013. Moreover, we intend to analyze the trend of repeated pregnancies and births by age groupings and potential macro-level confounders across two decades, with resulting trends perhaps reflecting the effectiveness of existing policies and programs in addressing this under-recognized adolescent health problem.

## Methods

### Population and sample

This study used the Philippine Demographic and Health Survey (DHS) from 1993, 1998, 2003, 2008, and 2013 which are cross-sectional surveys conducted every 5 years. This nationally representative survey involved a multi-stage sampling design up to the household level with enumeration areas distributed by region and type of residence using the most recent national census as its sampling frame. All women in the selected households which includes adolescents aged 15–19 years and young adults aged 20–24 years were interviewed using the Individual Woman’s Questionnaire. This survey therefore excludes adolescents aged below 15 years. As shown in Appendix, the majority of the survey sample belonged to these age brackets which we will refer to as adolescents for the succeeding parts of this paper.

### Outcome and socio-geographic measures

#### Repeated adolescent pregnancy/birth

An adolescent aged 15–19 years was considered as having experienced repeated pregnancy (RP) if she had experienced at least two pregnancies, including current pregnancies, which either resulted in a live birth and/or pregnancy loss. A case of repeated birth (RB) was defined as an adolescent with at least two live births. These definitions were adapted from related review papers [8] and the Centers for Disease Control and Prevention [7].

#### Year

Survey year was considered as a continuous variable in the analysis to measure the trend because of equal intervals between survey years. Thus, each unit increase in year variable translates to an actual five-year increase.

#### Age

Respondents were categorized by age into three and five groups. The three age groups include “15–18” which considers the legal age of consent (18) in the Philippines, “19–21” as the transition period, and “22–24” as young adults [19]. In sensitivity analysis we further subdivided age into five groups (i.e. “15–16”, “17–18”, “19–20”, “21–22”, and “22–24”) to analyze in detail the trends per age.

#### Socio-geographic variables

Region refers to the three main island groups: Luzon, Visayas, and Mindanao. We disaggregated and compared all estimates by region since each island group has unique geographical and cultural characteristics. Further disaggregation per administrative region was not pursued, as the number of administrative regions had increased during the 1998. Type of residence was either rural or urban area where the respondent resided at the time of the survey. Based on their household’s wealth score, adolescents were grouped into the household wealth quintiles “richest”, “richer”, “middle”, “poorer”, and “poorest” class.

### Analyses

We calculated the mean, standard deviation and prevalence rate of RP and RB per year per age group. RP prevalence was calculated by dividing the number of adolescents with RP and the number of adolescents who experienced at least one pregnancy (including those currently pregnant) multiplied by 100. RB prevalence on the other hand was calculated by dividing the number of adolescents with RB and the number of adolescents who experienced at least one livebirth multiplied by 100. Deformalized survey weights were applied while calculating the prevalence.

We used the *ptrendi* package in Stata13 to perform Cochran–Armitage tests to determine the prevalence trend per age group using the chi-square statistic and meeting the assumptions of an additive model. Cochran–Armitage test is a modified Pearson’s chi-square test which assesses the association between binary (i.e. RP and RB) and ordinal (i.e. year and age) categories. Multivariate logistic regression analysis with interaction effects for age (i.e. age groups using both three and five categories) and year was conducted while using repeated pregnancy and birth as binary outcome variables (i.e. yes or no). We measured the trend between two consecutive survey years to identify which periods had significant changes in prevalence. In addition, we analyzed trends using year and socio-geographic (i.e. region, type of residence, and wealth index) interaction per age group. For the purpose of this analysis, we used the three category age group as this was the only categorization which allowed a sufficient number of cases.

## Results

Among women aged 15–24 years with at least one pregnancy (*n* = 7091), a large proportion (53.3%) were found among the 22–24 year olds. Despite the small proportion of adolescents captured by the surveys, the proportion of 15–18 year olds reported in the survey has increased over time from 7.64% (*n* = 107) in 1993 to 15.55% (*n* = 213) in 2013 (*see* Table 1).

**Table 1:** Characteristics of the respondents

Characteristics	1993		1998		2003		2008		2013		TOTAL
	n	%	n	%	n	%	n	%	n	%	
Age (in 3 groups)											
15–18	107	7.64	124	9.74	128	9.54	165	11.97	213	12.55	**737**
19–21	460	32.86	449	35.27	429	31.97	479	34.74	580	34.18	**2397**
22–24	833	59.50	700	54.99	785	58.49	735	53.30	904	53.27	**3957**
Age (in 5 groups)											
15–16	14	1.00	19	1.49	21	1.56	33	2.39	45	2.65	**132**
17–18	93	6.64	105	8.25	107	7.97	132	9.57	168	9.90	**605**
19–20	268	19.14	268	21.05	269	20.04	300	21.75	352	20.74	**1457**
21–22	429	30.64	387	30.40	385	28.69	402	29.15	502	29.58	**2105**
23–24	596	42.57	494	38.81	560	41.73	512	37.13	630	37.12	**2792**
Region											
Luzon	685	48.93	523	41.08	728	54.25	699	50.69	870	51.27	**3505**
Visayas	275	19.64	244	19.17	187	13.93	229	16.61	232	13.67	**1167**
Mindanao	440	31.43	506	39.75	427	31.82	451	32.70	595	35.06	**2419**
Type of residence											
Urban	604	43.14	481	37.78	672	50.07	554	40.00	761	44.84	**3072**
Rural	796	56.86	792	62.22	670	49.93	825	59.83	936	55.16	**4019**
Wealth quintile											
Poorest	420	30.00	425	33.39	372	27.72	377	27.00	416	24.51	**2010**
Poorer	342	24.43	355	27.89	325	24.22	344	25.00	414	24.40	**1780**
Middle	292	20.86	210	16.50	272	20.27	256	18.56	389	22.92	**1419**
Richer	214	15.00	173	14.00	203	15.00	233	17.00	305	17.97	**1128**
Richest	132	9.43	110	8.64	170	12.67	169	12.26	173	10.19	**754**
**With at least 1 birth** ^**a**^	1260	90.00	1124	88.30	1181	88.00	1163	84.34	1471	86.68	**6199**
**With repeated pregnancy**	825	58.93	680	53.42	662	49.33	571	41.41	704	41.48	**3442**
**With repeated birth** ^**b**^	660	25.04	532	20.18	495	18.78	420	15.93	529	20.07	**2636**
**TOTAL** ^**+**^	**1400**	**100**	**1273**	**100**	**1342**	**100**	**1379**	**100**	**1697**	**100**	**7091**

Abbreviations: *n*-Number of respondents

^a^Birth pertains to livebirth; ^b^ Adolescents with at least 1 pregnancy

Data captured in bold are highly significant

### Trend analysis per age group

**Fig. 1:**
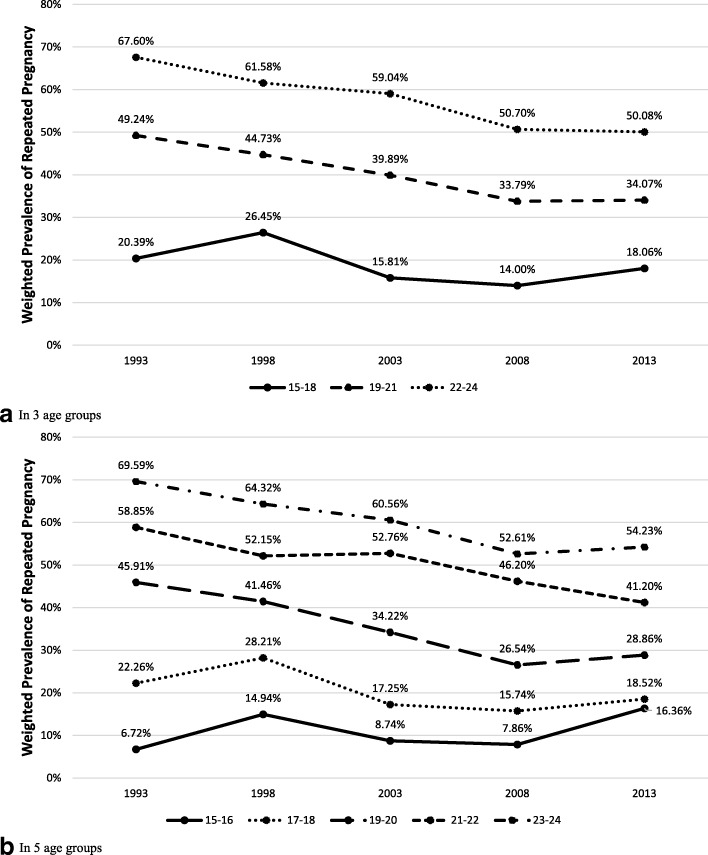
Prevalence trends of adolescents with repeated pregnancy in the Philippines from 1993 to 2013 by age group. Caption: This figure presents the weighted prevalence of repeated pregnancy using age groups with (**a**) three and (**b**) five categories. Groups using the three categories include 15–18 years old, 19–21 years old and 22–24 years old while the five categories including 15–16 years old, 17–18 years old, 19–20 years old, 21–22 years old and 23–24 years old, as represented by each line on the graphs. The x-axis is the survey year arranged in chronological order while the y-axis the weighted prevalence

**Fig. 2:**
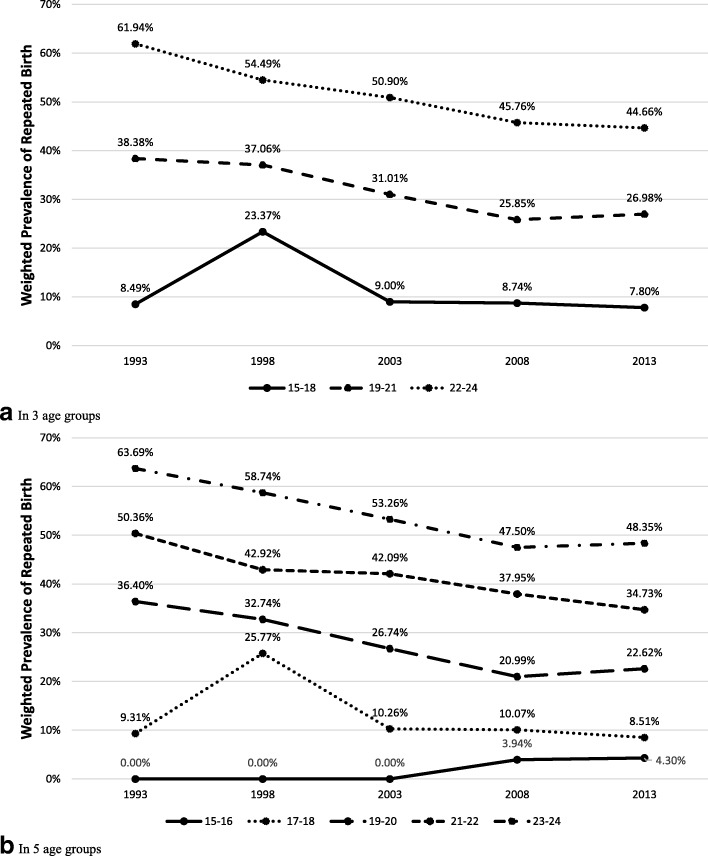
Prevalence trends of adolescents with repeated birth in the Philippines from 1993 to 2013 by age group. Caption: This figure presents the weighted prevalence of repeated birth using age groups with (**a**) three and (**b**) five categories. Groups using the three categories include 15–18 years old, 19–21 years old and 22–24 years old while the five categories including 15–16 years old, 17–18 years old, 19–20 years old, 21–22 years old and 23–24 years old, as represented by each line on the graphs. The x-axis is the survey year arranged in chronological order while the y-axis the weighted prevalence

Cochran–Armitage tests showed an overall decrease in the trend of RP (Chi2 = 127.60; *p* < 0.001) across 20 years among the 15–24 years old from a weighted RP prevalence (WtPrev_RP_) of 58.12% in 1993 to 40.58% in 2013. There was also a general RB (Chi2 = 100.90; *p* < 0.001) reduction from weighted RB prevalence (WtPrev_RB_) of 51.25% to 35.66%. However, within age groupings this decline was not observed among 15–18 years olds. In Fig. 1, we only found a slight decrease in RP prevalence from 20.39% in 1993 to 18.06% in 2013. RB prevalence also presented a minimal change with 0.69 decline among 15–18 and 0.80 decline among 17–18 years olds in this 20-year period (*see* Fig. 2). Further observations among 17–18 years olds showed a similar RP trend from 22.26 to 18.52%.

Similar results were found in the regression analysis. The RP trend among 15–18 year olds remained virtually unchanged across all surveys from 1993 to 2013 [Odds ratio (OR) =0.93; 95% Confidence interval (CI) =0.81–1.07]. There was a similar pattern of RB trend in this age group (OR = 0.87; CI = 0.72–1.06) following an apparent increase in prevalence from 1993 to 1998 (OR = 3.29; CI = 1.25–8.62). On the other hand, the older age groups showed a significant decline both for RP and RB with unadjusted ORs ranging from 0.83 to 0.87 (*see* Table 2). Analyses using five age categories showed no significant difference in the trends previously described. Trends among 15–16 and 17–18 year old adolescents remained unchanged, whereas a decreasing trend was apparent for those aged 19–20, 21–22 and 23–24.

**Table 2:** Trend analysis of repeated pregnancy and birth adolescents from 1993 to 2013 per age group

Year x Age Contrasts	Unadjusted Model			Adjusted Model ^b^		
	OR	CI	*p*-value	AOR	CI	*p*-value
*Repeated pregnancy*						
Age (in 3 groups)						
15–18	0.93	0.81–1.07	0.330	0.96	0.82–1.11	0.566
19-21^a^	0.85	0.80-0.90	< 0.001	0.86	0.81–0.92	< 0.001
22–24 ^a^	0.83	0.79–0.87	< 0.001	0.84	0.80–0.88	< 0.001
Age (in 5 groups)						
15–16	1.24	0.79–1.96	0.351	1.35	0.82–2.23	0.234
17–18	0.91	0.79–1.06	0.229	0.93	0.80–1.09	0.382
19–20 ^a^	0.82	0.76–0.89	< 0.001	0.82	0.76–0.90	< 0.001
21–22 ^a^	0.84	0.79–0.89	< 0.001	0.85	0.80–0.91	< 0.001
23–24 ^a^	0.84	0.80–0.89	< 0.001	0.85	0.80–0.90	< 0.001
*Repeated birth*						
Age (in 3 groups)						
15–18	0.87	0.72–1.06	0.181	0.90	0.73–1.10	0.311
19–21 ^a^	0.87	0.81–0.93	< 0.001	0.88	0.83–0.95	< 0.001
22–24 ^a^	0.84	0.80–0.88	< 0.001	0.85	0.82–0.89	< 0.001
Age (in 5 groups)						
15–16	2.15	0.54–8.57	0.275	2.47	0.54–11.46	0.245
17–18	0.87	0.72–1.07	0.186	0.90	0.73–1.11	0.316
19-20^a^	0.83	0.76-0.89	< 0.001	0.85	0.77–0.93	0.001
21–22 ^a^	0.86	0.80–0.92	< 0.001	0.87	0.82–0.94	< 0.001
23–24 ^a^	0.85	0.80–0.90	< 0.001	0.86	0.81–0.91	< 0.001

Abbreviations: OR-Odds ratio; CI-95% Confidence Interval

^a^Significant during Cochran test at 0.001 level; ^b^ Adjusted for region, type of residence and wealth quintile

Adjustments for regions, types of residence and wealth quintile suggested that the trends were not confounded by these factors across all age groups. Interestingly, wealth index was strongly associated with RP and RB as adolescents from the poorest quintile had shown higher odds in reference to richest quintile (OR_RP_ = 5.41, CI = 4.31–6.78; OR_RB_ = 5.36, CI = 4.17–6.89). Calculation of weighted prevalence confirmed this association with a WtPrev_RP_ of 59.60% and WtPrev_RB_ of 52.50%.

Change of prevalence between two consecutive survey years was also analyzed using the three age categories. We found that there was a decrease in RP prevalence among 15–18 from 1998 to 2003 (OR = 0.52; CI = 0.28–0.99), and among 22–24 from 1993 to 1998 (OR = 0.77; CI = 0.61–0.97) and 2003–2008 (OR = 0.71; CI = 0.58–0.88). A drop in RB prevalence was also found among 15–18 from 1998 to 2003 (OR = 0.32; OR = 0.13–0.81); and among 22–24 from 1993 to 1998 (OR = 0.74; CI = 0.58–0.93).

### Trend per socio-geographic variable per age group

The constant RP trend among 15–18 and the decreasing RP trend among 22–24 were found in all regions, types of residence and wealth quintiles (*see* Table 3*)*. On the other hand, the decline of RP decline among 19–21 was only consistent across regions and types of residence. Only the poorer households showed a 20-year reduction when compared to the other four quintiles.

**Table 3:** Trend analysis of repeated pregnancy and birth among adolescents per socio-geographic variable in each age group

(Year x Characteristics Contrasts)	Repeated pregnancy									Repeated birth								
	15–18 years old			19–21 years old			22–24 years old			15–18 years old			19–21 years old			22–24 years old		
	OR	CI	p	OR	CI	p	OR	CI	p	OR	CI	p	OR	CI	p	OR	CI	p
Region																		
Luzon	0.82	0.66–1.02	0.08	0.88	0.81–0.96	**0.004**	0.80	0.75–0.86	**< 0.001**	0.86	0.65–1.14	0.291	0.92	0.84–1.02	0.118	0.84	0.79–0.90	**< 0.001**
Visayas	1.09	0.74–1.61	0.65	0.74	0.64–0.86	**< 0.001**	0.84	0.73–0.93	**0.001**	0.94	0.57–1.55	0.801	0.77	0.65–0.91	**0.002**	0.81	0.72–0.91	**< 0.001**
Mindanao	1.04	0.82–1.30	0.763	0.85	0.77–0.94	**0.001**	0.91	0.84–0.93	**0.017**	0.86	0.63–1.18	0.358	0.82	0.74–0.91	**< 0.001**	0.87	0.80–0.94	**0.001**
Type of residence																		
Urban	0.88	0.69–1.11	0.282	0.88	0.80–0.96	**0.006**	0.80	0.74–0.86	**< 0.001**	0.89	0.62–1.27	0.528	0.95	0.85–1.05	0.288	0.81	0.75–0.87	**< 0.001**
Rural	0.98	0.82–1.18	0.85	0.83	0.76–0.90	**< 0.001**	0.86	0.81–0.92	**< 0.001**	0.88	0.69–1.13	0.307	0.81	0.74–0.88	**< 0.001**	0.87	0.82–0.93	**< 0.011**
Wealth quintile																		
Poorest	0.95	0.75–1.20	0.677	0.9	0.81–1.00	0.056	0.90	0.81–0.99	**0.03**	0.82	0.59–1.14	0.239	0.88	0.79–0.98	**0.018**	0.90	0.82–1.00	**0.041**
Poorer	0.90	0.69–1.19	0.463	0.77	0.69–0.86	**< 0.001**	0.87	0.79–0.96	**0.005**	0.97	0.70–1.33	0.833	0.80	0.70–0.91	**0.001**	0.89	0.81–0.98	**0.018**
Middle	0.98	0.71–1.36	0.905	0.92	0.80–1.05	0.202	0.87	0.79–0.95	**0.003**	0.85	0.51–1.44	0.551	0.93	0.79–1.10	0.379	0.87	0.79–0.96	**0.004**
Richer	1.01	0.66–1.53	0.97	0.89	0.75–1.05	0.16	0.82	0.72–0.91	**< 0.001**	1.00	0.53–1.88	0.993	0.96	0.79–1.17	0.685	0.79	0.70–0.89	**< 0.001**
Richest	0.69	0.27–1.80	0.45	0.89	0.70–1.11	0.298	0.68	0.59–0.79	**< 0.001**	NC			0.99	0.76–1.29	0.927	0.73	0.62–0.86	**< 0.001**
**Adjusted Wald Test**	**F**	**p**		**F**	**p**		**F**	**p**		**F**	**p**		**F**	**p**		**F**	**p**	
Region																		
Luzon vs. Visayas	1.57	0.211		3.69	0.054		0.18	0.668		0.09	0.766		3.58	0.059		0.30	0.585	
Luzon vs. Mindanao	2.04	0.154		0.30	0.582		6.01	**0.014**		0.00	0.967		2.56	0.110		0.47	0.493	
Visayas vs. Mindanao	0.06	0.812		2.07	0.151		1.86	0.173		0.08	0.782		0.47	0.491		1.06	0.302	
Type of residence																		
Urban vs. Rural	0.54	0.462		0.94	0.33		2.62	0.106		0.00	0.951		5.21	**0.023**		2.34	0.127	
Wealth quintile																		
Poorest vs. Poorer	0.07	0.787		4.29	**0.039**		0.13	0.718		0.49	0.485		1.22	0.289		0.04	0.844	
Poorest vs. Middle	0.02	0.876		0.03	0.87		0.20	0.656		0.01	0.907		0.31	0.58		0.35	0.55	
Poorest vs. Richer	0.07	0.797		0.03	0.853		1.43	0.232		0.28	0.595		0.60	0.44		2.74	0.098	
Poorest vs. Richest	0.40	0.528		0.02	0.883		9.18	**0.003**		NA			0.65	0.419		5.00	**0.023**	
Poorer vs. Middle	0.15	0.70		3.64	0.057		0.01	0.933		0.16	0.689		1.90	0.169		0.14	0.705	
Poorer vs. Richer	0.18	0.668		1.87	0.172		0.83	0.363		0.01	0.93		2.27	0.133		2.37	0.124	
Poorer vs. Richest	0.28	0.599		1.14	0.285		7.86	**0.005**		NA			1.96	0.162		4.39	**0.036**	
Middle vs. Richer	0.01	0.915		0.09	0.763		0.75	0.387		0.14	0.709		0.06	0.801		1.47	0.226	
Middle vs. Richest	0.45	0.504		0.06	0.807		7.63	**0.006**		NA			0.15	0.70		3.25	0.072	
Richer vs. Richest	0.51	0.475		0.00	0.999		3.78	0.052		NA			0.03	0.866		0.65	0.42	

Abbreviations: OR-Odds ratio; CI-95% Confidence Interval; p-*p* value; F-F statistic; NC-No cases; NA-Not applicable; OR-Odds ratio

Data captured in bold are highly significant

**Fig. 3:**
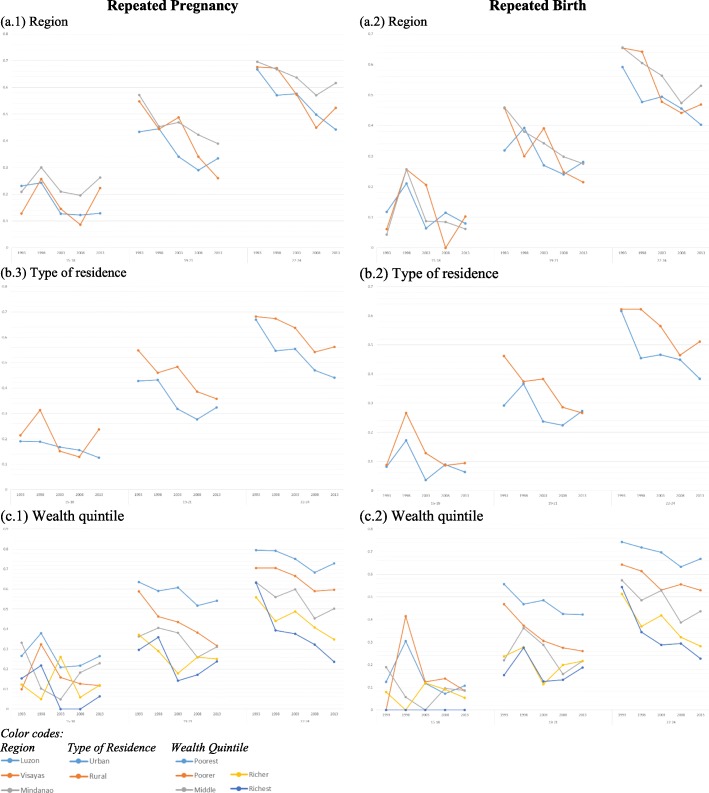
Prevalence trend of repeated pregnancies and births among adolescents per socio-geographic variable in each age group. Caption: This figure presents the trend of the weighted prevalence of repeated pregnancies and births in each of the socio-graphic variable using the three age categories: 15–18 years old, 19–21 years old and 22–24 years old. The left column presents the weighted prevalence of repeated pregnancy while the right column presents repeated birth. In each graph, the x-axis is the survey year arranged in chronological order while the y-axis the weighted prevalence. The color of each line represents a category of each socio-geographic variable as shown at the bottom of the graph

A similar pattern was observed for RB trend among those aged between 15 and 18 and 22–24. Unlike RP, the trend for RB among 19–21 year olds was inconsistent across the three socio-geographic variables. The decreasing trend was only found in Visayas and Mindanao region, rural communities, and poor wealth quintiles *(see* Fig. 3*)*.

In each age group, we also conducted adjusted Wald tests to measure the difference of trend estimates between the categories of each socio-geographic variable. No differences were observed for 15–18. For 19–21, differences were only found between the RP trend estimates of poorest and poorer quintiles, and between the RB trend estimates rural and urban communities. For 22–24, differences between the trend estimates of poorest and richest, and between poorer and richest were found both for RP and RB.

## Discussions

Despite the declining trends of RP and RB in older age groups, the prevalence among adolescents younger than 18 years showed no decrease across 20 years of data, remaining stable across all regions, types of residence, and wealth quintiles. The prevalence was high with approximately one in every five adolescents aged 15–18 years with a history of pregnancy experiencing RP while one in every ten of those who had a livebirth experienced RB.

While the decreasing RP and RB trend among young adults can likely be attributed to their improved contraceptive use [20] and awareness of and participation in family planning (FP) strategies [3, 21]. The unchanged trend among adolescents may result from the unique socio-cultural characteristics and FP policies in the Philippines, wherein adolescents are prevented from accessing FP services, even after their first pregnancy. One of the possible explanations for this finding is that the strong influence of the Catholic church at the local level may have affected the health seeking behavior and the implementation of reproductive health programs among adolescents [22, 23].

Unclear and restricted health and health-related policies for adolescent mothers may also play a role. The initial adolescent health policy in the Philippines [24], which aimed to reduce unwanted pregnancies and provide adolescent-friendly health services, did not include strategies for dealing with the prevention of secondary pregnancies [25, 26]. This may have led to adolescents being discouraged to access essential health information and use birth control methods [23, 27].

Despite emphasizing the importance of health promotion and behavioral change, a recently introduced national law (Responsible Parenthood and Reproductive Health Act of 2012 or RH Law) and framework [4], did not embrace specific programmatic actions to address RP. The RH Law still prevents minors (i.e. below 18 years old) from accessing modern methods of contraception without parental consent and does not exempt adolescent mothers and adolescents who experienced miscarriage [28]. This policy restriction has already been found as a deterrent for adolescents to access contraceptives and counselling services in a review of evidence from 16 developing countries [29]. This study suggests that despite the availability of contraception, most of these developing countries retain barriers and restrictions towards the use of birth control methods, particularly among unmarried adolescents. In the context of this social and political environment, the RP/RB trends showed in this paper can be expected to continue for several years to come not only in the Philippines but also in other developing countries.

The role and reach of secondary prevention programs must be clarified due to the limited access to appropriate postnatal services (e.g. contraception, counselling, and educational support) for adolescent mothers. Health workers may also need to be trained to address the unique psychosocial characteristics and support the challenging developmental transition of very young mothers by enhancing adolescents’ readiness and decision-making abilities to delay another pregnancy and/or use modern family planning methods. Given the high rate of unmet need for modern contraception among married adolescents [21], policy initiatives/reforms such as providing exemption on contraception to adolescent mothers may be needed to achieve a reduction in the trend seen in this paper.

Our findings also suggest that prevention programs aimed at those from the poorest quintile may be warranted due to the high RP/RB prevalence among this group. In the Philippines and other low- and middle-income countries (LMICs), attempts to reach out to households from the poorest sector have been undertaken through the Conditional Cash Transfer (CCT) Program [30, 31]. For example, the CCT program in Mexico has been found to indirectly reduce adolescent pregnancy and increase contraceptive use among adolescents and young adults [31]. The potential of cash incentive schemes can also be used as an opportunity to monitor and provide prevention programs to adolescent mothers, particularly within 24 months after their first pregnancy [10].

Our study uniquely explores the status of repeated pregnancy and birth in LMICs in the Asia-pacific Region. Most published reports on this topic are primarily from the USA, Europe, and Australia [32]. Of the few reports identified from LMICs, many used birth order (i.e. 2nd order or higher) and a different denominator (i.e. total number of adolescents) in the computation of prevalence. Despite the availability of possible data sources among LMICs [33], few studies have attempted to look specifically at the distribution of adolescents and young adults with RP/RB. Most of the reports available may include vital statistics which is limited to those only with livebirths and does not necessarily account for previous unsuccessful pregnancies.

By placing RP as an issue of crucial importance to the public health especially of LMICs, our paper makes a significant contribution to the literature calling for improvement of sexual and reproductive health of adolescents. The Global Strategy for Adolescent Health for 2030 recognized childbirth and pregnancy complications as one of the two leading causes of death among 15–19 year old girls [34]—addressing RP would help to reduce this. The absence of a reduction in RP trend over 20 years that we identified, signals the need for secondary prevention programs in line with WHO recommendations [35].

This study finds strength in our use of nationally-representative individual datasets instead of aggregate estimates. This prevents the risk of producing results affected by the ecological fallacy, particularly in the analysis of year-age interaction. Furthermore, we were able to perform more thorough analyses such as the adjustment of trend estimates for confounders (i.e. wealth quintile, region, and type of residence).

### Limitations

Our study also has limitations. Recall bias and under-reporting are likely to produce bias in any surveys covering information of a sensitive nature. Insufficient record validation is common across the DHS surveys from all countries. However, the DHS’ survey procedure enables cross-checking through repeated questions during the interview to reduce the effect of this validation issue. Additionally, our findings may not be comparable to longitudinal studies from developed countries that defined RP as an adolescent who became pregnant within 12–24 months of her first pregnancy/ delivery.

### Future research

In addition to cross-sectional analyses that measure RP prevalence, epidemiological investigations are needed to explore the causes and outcomes of RP. Studies conducted in LMICs may identify different associations and dynamics due to the psychosocial and cultural characteristics of and attitudes towards adolescent mothers in these countries. This type of study not only directs the development of specialized perinatal care, and psychosocial and welfare support but also places priority on those adolescents with RP.

A multi-country analysis would also be beneficial in obtaining a broader RP status especially in countries with similar characteristics. This would help international organizations to implement immediate action for RP in a global approach and prioritize countries with a high RP burden. Additionally, projection of RP prevalence at least until 2030 using country-level determinants such as contraceptive prevalence, poverty, literacy, and maternal-child mortality rates, may facilitate target setting for this potential adolescent reproductive health indicator.

## Conclusion

There is a constant trend of one in every five adolescent mothers in the Philippines experiencing repeated pregnancy from 1993 to 2013 (across all regions, type of residence, and socio-economic status). These findings indicate the need for secondary prevention programs, particularly among the poorest households. Epidemiological investigations are also necessary to explore the causes and impacts of repeated pregnancy on maternal, child, and neonatal health in the Philippines and other low- and middle-income countries.

## Appendix

**Table 4:** Characteristics of National Demographic and Health Survey (NDHS) Philippines from 1993 to 2013

Characteristics	Year				
	2013	2008	2003	1998	1993
Sampling					
Frame	2010 Census	2000 Census	2000 Census	1995 Census	1990 Census
Design	Stratified Two-stage *First stage:* Systematic selection of 800 Enumeration Areas (EAs) distributed by region (17) and type of residence (rural, urban) *Second stage:* Systematic selection of 20 housing units per EA (maximum of 3 households per unit)	Stratified Three-stage *First stage:* Selection of PSUs *Second stage:* Selection of 794 EAs *Third stage:* Selection of 17 housing units per EA through systematic random sampling (maximum of 3 households per unit)	Stratified Three-stage Cluster *First stage:* Selection of 819 PSUs *Second Stage:* Selection of EAs per PSU *Third stage:* Systematic selection of 17 households per EA	Stratified Two-stage *First stage:* Selection of 752 EAs on 16 regions *Second Stage:* Systematic selection of households in urban while cluster sampling among rural areas	Stratified Two-stage *First stage:* Selection of 750 PSUs distributed among 14 regions Second stage: Selection of 20 households per PSU
Response Rates					
Households	99.2	99.3	99.1	98.7	99.2
Women (15–49 years old)	98.3	98.3	97.8	97.2	98
Sample Size					
Households	14,804	12,469	12,586	12,407	12,995
n of Women (15–19 years old) Weighted % distribution	3261 20%	2766 20.2%	2646 19.4%	2949 20.9%	3139 21%
n of Women (15–24 years old)	6070 37.3%	4909 36%	4860 35.6%	5190 37.3%	5741 38.6%

## Abbreviations

CCT: Conditional Cash Transfer
CI: 95% Confidence Interval
DHS: Demographic and Health Survey
FP: Family planning
LMICs: Low- and middle-income countries
OR: Odds ratio
RB: Repeated birth
RP: Repeated Pregnancy
WHO: World Health Organization
WtPrevRB: Weighted RB prevalence
WtPrevRP: Weighted RP prevalence
